# Comparison of photon volumetric modulated arc therapy, intensity-modulated proton therapy, and intensity-modulated carbon ion therapy for delivery of hypo-fractionated thoracic radiotherapy

**DOI:** 10.1186/s13014-017-0866-0

**Published:** 2017-08-15

**Authors:** Alexander Chi, Lien-Chun Lin, Sijin Wen, Haijuan Yan, Wen-Chien Hsi

**Affiliations:** 1 Shanghai Proton and Heavy Ion Center, Shanghai, China; 2 Department of Biostatistics, West Virginia University, Morgantown, WV USA

**Keywords:** NSCLC, VMAT, IMPT, IMCIT, Proton, Carbon ion

## Abstract

**Purpose:**

The aim of the present study was to compare the dose distribution generated from photon volumetric modulated arc therapy (VMAT), intensity modulated proton therapy (IMPT), and intensity modulated carbon ion therapy (IMCIT) in the delivery of hypo-fractionated thoracic radiotherapy.

**Methods and materials:**

Ten selected patients who underwent thoracic particle therapy between 2015 and 2016 were re-planned to receive a relative biological effectiveness (RBE) weighted dose of 60 Gy (i.e., GyE) in 15 fractions delivered with VMAT, IMPT, or IMCIT with the same optimization criteria. Treatment plans were then compared.

**Results:**

There were no significant differences in target volume dose coverage or dose conformity, except improved D_95_ was found with IMCIT compared with VMAT (*p* = 0.01), and IMCIT was significantly better than IMPT in all target volume dose parameters. Particle therapy led to more prominent lung sparing at low doses, and this result was most prominent with IMCIT (*p* < 0.05). Improved sparing of other thoracic organs at risk (OARs) was observed with particle therapy, and IMCIT further lowered the D_1cc_ and D_5cc_ for major blood vessels, as compared with IMPT (*p* = 0.01).

**Conclusion:**

Although it was comparable to VMAT, IMCIT led to significantly better tumor target dose coverage and conformity than did IMPT. Particle therapy, compared with VMAT, improved thoracic OAR sparing. IMCIT, compared with IMPT, may further improve normal lung and major blood vessel sparing under limited respiratory motion.

**Electronic supplementary material:**

The online version of this article (doi:10.1186/s13014-017-0866-0) contains supplementary material, which is available to authorized users.

## Background

Early-stage non-small cell lung cancer (NSCLC) and lung metastases have been treated with stereotactic body radiation therapy (SBRT) with excellent clinical outcome [1–4]. Locally advanced NSCLC has traditionally been treated with conventionally fractionated concurrent chemo-radiation [5]. However, dose escalation with conventional fractionation has not demonstrated any clinical advantage in any phase III randomized controlled trials [6]. As a result, alternative radio-therapeutic strategies are being sought. Given the clinical efficacy that has been observed with hypo-fractionated dose schedules, such as SBRT, this approach has also been increasingly considered as a treatment strategy for locally advanced and selected stage IV NSCLC in recent years [7–9].

Excellent dose distribution can be achieved with image-guided and intensity-modulated radiotherapy (IG-IMRT) delivered with volumetric modulated arc therapy (VMAT) in the thorax [10, 11]. As a result, VMAT has been quickly adopted in clinical settings to deliver thoracic radiotherapy [12]. However, sparing thoracic organs at risk (OARs) while maintaining adequate target volume dose coverage with VMAT remains challenging in selected patients. On the contrary, particle therapy (PT), which includes proton and heavy ion therapies, may have an advantage over VMAT in OAR sparing. This improvement is due to PT’s physical properties, which allow for better normal tissue protection, while heavy ions’ increased radiobiological effectiveness (RBE) increases the tumorcidal effect of radiotherapy over photons [13, 14]. PT’s dosimetric advantages have been shown in multiple studies [15–22]. Given these advantages, PT is increasingly being considered for the delivery of hypo-fractionated thoracic radiotherapy [23, 24]. To date, PT has been mostly delivered with passive scattering systems. With further improvements in technology, intensity modulation of particle beams with active beam scanning has been developed for better dose conformity and control of the OAR dose. However, little is known about how active scanning beams compare with VMAT in dose distribution in the treatment of lung tumors. In this study, we explored the dosimetric characteristics of intensity-modulated proton therapy (IMPT) and carbon ion therapy (IMCIT) with raster scanning beams in the delivery of hypo-fractionated thoracic radiotherapy in comparison to VMAT under limited respiratory motion.

## Methods

### Patients, tumors and treatment characteristics

Ten selected patients who underwent either IMPT or IMCIT between 2015 and 2016 were re-planned. Among them, 9 patients had stage I - III NSCLC, and 1 patient had a single lung metastasis from adenoid cystic carcinoma of the head and neck (Table 1). All patients were simulated head first in prone or supine positions. Each patient was immobilized with Vac-Lok™ cushions (CIVCO Medical Solutions, IA, USA). During CT simulation, free-breathing CT and 4D CT (divided evenly over 10 phases of the respiratory cycle) were obtained with 3-mm slice thickness to account for respiratory motion. All patients were planned for gated treatments with a pressure sensor-based motion management system (AZ-773 V, Anzai Medical, Co., Ltd., Tokyo, Japan). The gating window (30–40% duty cycle) was selected to keep the respiratory motion to no more than 5 mm cranio-caudally (Table 1). This motion limitation was accomplished through visual inspection of the 4D CT's to select the phases from the end of expiration to the beginning of inspiration that were confined within a cranio-caudal tumor motion amplitude of ≤5 mm. The primary gross tumor volume (GTV) was delineated at the lung window level, and any nodal metastases and soft tissue extensions were delineated at the soft tissue window level on the maximum exhalation CT. To account for tumor motion, an iGTV was created with the maximal intensity projection (MIP) generated from CTs of the selected respiratory phases. The clinical target volume (CTV) was the iGTV with an 8-mm expansion. The planning target volume (PTV) was the CTV with a 5-mm expansion to account for set up errors and residual tumor motion. The CTV and PTV were created on the average CT of the selected respiratory phases. Particular attention was paid to avoid overlapping of any target volumes with the OARs. The lungs, esophagus, spinal cord, and the heart were contoured for each patient on the average CT. The major blood vessels and major airway were contoured only when they were adjacent to the PTV. Target and OAR volume delineation was performed with MIM v6.5.9 software (MIM software Inc., Cleveland, OH, USA).

**Table 1:** Patient, tumor, and treatment characteristics

n	Age	Gender	Diagnosis	TNM (AJCC 8th edition)	Tumor location	Total PTV (cc)	Rx position	Rapid arc angle (2 arcs)	Couch angles (3F)	Gating window	Motion amplitude^a^ (mm)
1	58	M	NSCLC	T4, N0, M0	LUL	341.83	Prone	0–181	210, 260, 300	40%Ex - 20%In	4.5
2	46	M	NSCLC	T4, N3, M0	RUL	220.29	Prone	0–181	180, 230, 320	40%Ex - 20%In	5
3	64	M	NSCLC	T1a2, N0, M0	LUL	67.51	Prone	50–260	185, 225, 265	40%Ex - 20%In	2
4	79	M	Lung metastasis	M1 (6.99 cm)	RLL	521.07	Prone	0–179	10, 290, 330	20%Ex - 20%In	4
5	73	M	NSCLC	T4, N3, M0	LUL	358.21	Prone	0–181	175, 210, 330	40%Ex - 20%In	4.5
6	71	M	NSCLC	T2a, N0, M0	LUL	118.84	Supine	0–179	240, 300, 350	40%Ex - 20%In	2
7	66	M	NSCLC	T4, N3, M0	RUL	566.97	Prone	0–181	200, 240, 330	40%Ex - 20%In	3
8	60	M	NSCLC	L: T3; R: T3	LLL, RUL	L: 50.21; R: 102.88	Prone	L: 0–181; R: 80–320	L: 180, 225, 270 R: 0, 240, 290	20%Ex - 20%In	5 2
9	72	M	NSCLC	T3, N0, M0	RLL	40.76	Prone	0–179	0, 270, 315	40%Ex - 20%In	3
10	77	M	NSCLC	T3, N0, M0	RLL	146.47	Prone	0–179	180, 250, 330	20%Ex - 20%In	4

*Abbreviations*: *3F* 3 fields, *Ex* exhale, *In* inhale, ^a^cranio-caudal

All plans prescribed 60 Gy/GyE (relative biological effectiveness, RBE, weighted dose for IMPT and IMCIT) delivered in 15 daily fractions to the PTV with heterogeneity corrections. For VMAT, a 1.0-Gy physical dose is identical to 1.0 GyE. For IMPT, a 1.0-Gy physical dose is equal to a constant factor of 1.1 GyE at all positions. However, a 1.0-Gy physical dose for IMCIT was scaled to biological doses according to the LEM-1 model. Raster-continuous spot scanning without stopping between spots was delivered with dynamic intensity control to decrease the time required to deliver large doses (resulting in a maximum of 1–2 ms per spot). In our beam delivery system, the beam intensity could be dynamically changed between spots according to the total number of particles required for each spot. The change in beam intensity could be performed within 100 μs. Because our beam delivery system was calibrated to the number of particles per unit dose Gy, 13 levels of intensity for both carbon-ion and proton beams could be used clinically (1.3 × 10^6^ to 6.5 × 10^7^/s for carbon ions and 5.0 × 10^7^ to 2.6 × 10^9^/s for protons). However, only the final 6 highest levels (within ¼ of the maximum intensities) were used for actual treatment. The average dose rate at the center of each spot during beam delivery was 2.0 to 10.0 Gy/s or 12.0 to 60.0 Gy/min for both carbon-ion and proton beams. The dose delivery time of each energy-layer was typically within 1.0 s. With a typical energy-layer switching time of approximately 4–5 s for both carbon-ion and proton beams, the doses for spots in each energy layer were typically delivered within a 5-s breathing cycle.

The plans were optimized to have ≥ 90% of the PTV receiving 100% of the prescription dose and ≥ 95% of the PTV receiving ≥ 95% of the prescription dose. OAR dose constraints were adopted from previous studies on hypo-fractionated thoracic radiotherapy [8, 23]. PTV coverage took precedence over OAR sparing in all plans, which were optimized under the same set of planning criteria.

### Photon planning

VMAT plans were generated with Rapid Arc (RA) using the Eclipse Treatment Planning System V.11 (Varian Medical Systems. Palo Alto, CA, USA) with 6 MV photons. Plans were generated with co-planar partial arcs to spare as much contralateral lung as possible. The arc angle and collimator setting were based on the target size, shape, and location. Then, 2-arc plans were created for each case at dynamic multi-leaf collimator motion, variable dose rate (max: 600 MU/min), and variable gantry rotation speed to generate a modulated dose distribution. The arc length varied from 0° to 320°. All plans were optimized using the Progressive Resolution Optimizer (PRO, v11.0.31), and the volume dose was calculated with the Anisotropic Analytical Algorithm (AAA, V11.0.31) with a 2.5-mm grid size resolution.

### PT planning (IMPT & IMCIT)

IMPT and IMCIT plans were generated with multi-field optimization (MFO) by using the Syngo® PT Planning System v10 (Siemens. Erlangen, Germany). The effective dose calculation model (Local Effect Model, LEM) was used for IMCIT dose calculation [25, 26], and IMPT dose calculation was performed with a constant RBE factor of 1.1. Plan optimization was done on average CT's with iGTV density overridden (to the density of muscle) [27]. All plans were implemented in an oblique (45°) beam-line room with a raster scanning delivery technique. With the fixed 45° beam-line, three different angles of the robotic couch were selected to perform non-coplanar optimization. Angle selection depended on the tumor location and size, beam path according to OAR location, and the innate restriction of the robotic couch to avoid any unnecessary dose and to evade strong density heterogeneities upon the beam’s entry. Beam directions traversing patient support and immobilization were avoided. The setting of the focal spot size for the lateral full-width half-maximum (FWHM) of the scanning beam was 8 mm, and the longitudinal beam spot range step was 3 mm. Considering the calculation time and accuracy, we used 3 mm as the dose grid resolution for both proton and carbon ion treatment plans. Finally, the virtual target expansion was specified as 3 mm in lateral, proximal, and distal directions to allow optimal placement of additional raster spots outside the PTV within the expansion volume to satisfy target coverage requirements. The estimated treatment delivery times (median) for carbon-ion and proton plans were 736 (281–1009) seconds and 514.5 (276–703.5) seconds, respectively.

### Plan comparison

All plans were transferred into MIM software for direct comparison of the dose parameters obtained from the VMAT, IMPT, and IMCIT plans. OAR dose parameters, including the mean lung dose (MLD), the volume of the normal lung receiving 5 Gy, 10 Gy, and 20 Gy (V_5_, V_10_, and V_20_, respectively) for the total lung (both lungs – iGTV), the ipsilateral lung and the contralateral lung, the heart’s mean dose (HMD), V_5_, V_15_, and V_30_, the maximum dose (D_max_), doses to 0.01 cm^3^, and 1 cm^3^ (D_0.01cm_^3^ and D_1cc_, respectively) of the esophagus, spinal cord, major vessels and the major airways, were compared. For the PTV, the dose covering 95% and 99% of the PTV (D_95_, D_99_), the percentage of PTV receiving ≥95% of the prescribed dose (V_95_), D_min_, D_max_, D_mean_, the homogeneity index (HI), and the conformity index (CI) were compared among the 3 treatment techniques. The CI and HI were defined as [20]:

1 $$ \mathrm{CI}={\mathrm{V}}_{95\%\mathrm{Rx}}/{\mathrm{V}}_{\mathrm{PTV}} $$

2 $$ \mathrm{HI}={\mathrm{D}}_{\mathrm{max}}/{\mathrm{D}}_{\mathrm{Rx}} $$

where V_95%Rx_ and V_PTV_ represent the volume of tissue  receiving ≥ 95% of the prescribed dose and the volume of the PTV, respectively. D_max_ and D_Rx_ represent the maximum dose within the PTV and the prescribed dose, respectively. The new Conformity Index (nCI) was also assessed to evaluate the degree to which the prescribed isodose volume conforms to the target volume [28].

3 $$ \mathrm{nCI}=\left[\left(\mathrm{treatment}\  \mathrm{volume}\right)\times \left(\mathrm{prescription}\  \mathrm{isodose}\  \mathrm{volume}\right)\right]/{\left(\mathrm{volume}\  \mathrm{of}\  \mathrm{the}\  \mathrm{target}\  \mathrm{covered}\ \mathrm{by}\ \mathrm{the}\  \mathrm{prescription}\  \mathrm{isodose}\  \mathrm{volume}\right)}^2 $$

### Dose evaluation with recalculation on selected 4D phases within the gating window

Gating has been applied in recognition of the effect of the interplay on dose due to variations in the temporal relationship between dynamic beam scanning and target motion, as described in previous studies [29, 30]. By applying gating to limit the target movement to within 5 mm, dose heterogeneity was found to be within the clinically acceptable range of 3% to 5% at our institution (Additional file 1). Without the ability to fully account for this interplay, we focused mainly on the effect of uncertainties in dose due to anatomical changes within the gating window of 5 mm by retrospectively recalculating the IMCT and IMPT plans using selected 4D CT phases within the gating window. The spot pattern and doses obtained in the 3D plan were used to recalculate the doses on selected CT phases. Dose distributions of all recalculated 4D phases were accumulated to the reference CT by utilizing the deformable registration workflow provided by MIM 6.5.9. Selected dose parameters from the corresponding DVH’s were then extracted and compared with those from the original 3D plans.

### Statistical analysis

The primary objective of this study was the dosimetric comparison of VMAT, IMPT and IMCIT for delivery of hypo-fractionated thoracic radiotherapy. The dose parameters were obtained from treatment plans for the three radio-therapeutic modalities for each patient and were analyzed as continuous variables. A sample size of 10 patients had 80% power to detect one standard deviation difference by using a two-sided paired t-test at a 0.05 significance level. Descriptive statistics were used to summarize patient data, including OAR dose parameters and target volume dose coverage parameters. Categorical data were described using contingency tables including counts and percentages; continuous variables were summarized with descriptive statistical measures (i.e., mean (± s.d.)). The Wilcoxon signed rank test was used to assess dose parameters or target volume dose coverage as paired data (within same patients but different radio-therapeutic modalities) without the assumption of a normal distribution. A *p*-value <0.05 was considered to be statistically significant. Statistical analyses were performed using SAS 9.2 and R software, version R 3.1.3 (SAS Institute Inc., Cary, NC, USA).

## Results

### Target volume dose distribution

No statistically significant differences in any pertinent PTV dose coverage parameters, with the exception of D_95_, was observed between VMAT and IMCIT (Table 2). In contrast, statistically significant differences in all parameters favoring fixed-beam IMCIT over fixed-beam IMPT were observed (*p* < 0.05). Although no statistically significant differences in V_95_, D_95_, and D_99_ were observed between VMAT and fixed-beam IMPT, VMAT led to significantly lower D_max_, CI, HI, and nCI (*p* < 0.05).

**Table 2:** Target volume dose distribution

Parameters	VMAT_1_	IMPT_2_	IMCIT_3_		*p* value	
	Mean (SD)			*p*_1,2_	*p*_2,3_	*p*_1,3_
V_95_	100% (0)	99% (1%)	100% (0)	1.00	0.02	0.46
D_95_^a^	59.57 (0.40)	59.63 (0.67)	59.85 (0.47)	0.17	0.04	0.01
D_99_^a^	58.00 (0.75)	57.99 (1.25)	58.38 (0.81)	0.46	0.02	0.21
D_max_^a^	64.87 (0.92)	65.98 (1.14)	65.25 (0.77)	0.04	0.01	0.41
CI	1.25 (0.10)	1.37 (0.11)	1.30 (0.09)	0.01	0.00	0.11
HI	1.08 (0.02)	1.10 (0.02)	1.09 (0.01)	0.03	0.02	0.20
nCI	1.47 (0.80)	1.53 (0.78)	1.47 (0.77)	0.01	0.00	0.79

*p*_1,2_ is VMAT vs. IMPT; *p*_2,3_ is IMPT vs. IMCIT; *p*_1,3_ is VMAT vs. IMCIT. ^a^Dose in Gy

### Dose to the normal lungs

**Fig. 1:**
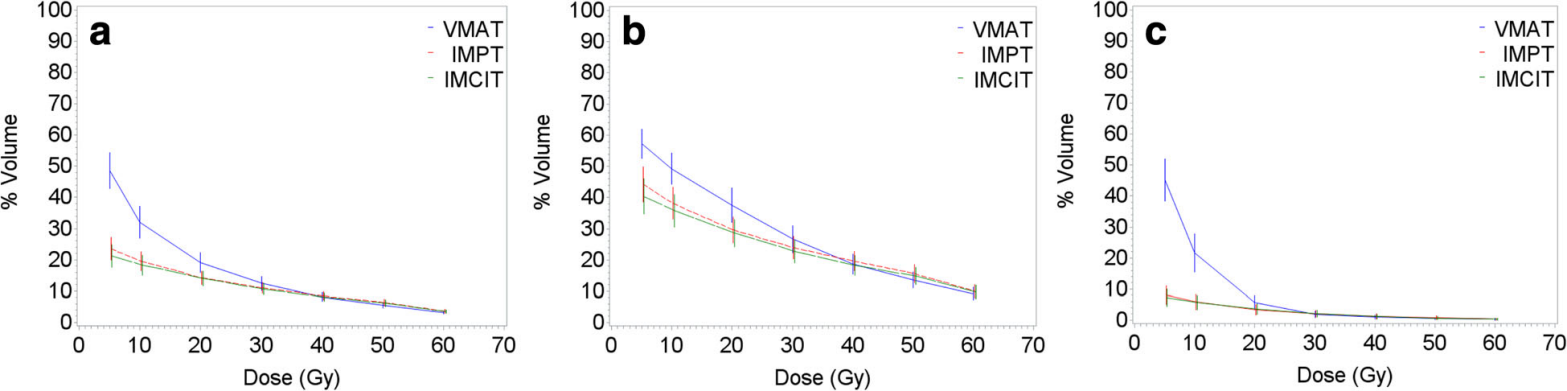
Dose distribution for normal lungs. **a** Dose distribution for the total lung (both lungs – iGTV). **b** Dose distribution for the ipsilateral lung. **c** Dose distribution for the contralateral lung

The volumes of normal lung tissue receiving low doses were significantly smaller for PT (Additional file 2: Table S1; Fig. 1). However, the V_50_’s for the total and ipsilateral lung were slightly, but significantly, higher for PT than VMAT (*p* < 0.05). A trend toward an increased ipsilateral lung V_60_ was also observed with IMPT compared with VMAT (*p* = 0.06). PT led to significantly lower V_5_, V_10_, and V_20_ for the total, ipsilateral, and contralateral lung than did photon VMAT (*p* < 0.05, Table 3). The contralateral lung’s MLD was significantly lower with PT (*p* < 0.05). The MLD for the total lung was similar between VMAT, IMPT, and IMCIT, whereas a lower MLD was observed for IMCIT compared with VMAT, with a trend toward statistical significance (*p* = 0.07). In addition, IMCIT further decreased the V_5_ and V_10_ for the total and ipsilateral lung from that achieved with IMPT (*p* < 0.05). Additionally, lower ipsilateral lung V_30_ for IMCIT compared with IMPT exhibited a trend toward statistical significance (*p* = 0.09).

**Table 3:** Normal lung dose volume parameters

Parameters	VMAT_1_	IMPT_2_	IMCIT_3_		*p* value	
	Mean (SD)			*p*_1,2_	*p*_2,3_	*p*_1,3_
Total lung - iGTV						
MLD (Gy)	11.38 (4.67)	7.64 (3.53)	7.25 (3.67)	0.11	0.97	0.07
V_5_	49% (18%)	24% (11%)	21% (12%)	0.00	0.00	0.00
V_10_	32% (16%)	20% (10%)	18% (10%)	0.00	0.04	0.00
V_20_	19% (10%)	14% (7%)	14% (8%)	0.00	0.56	0.00
Ipsilateral lung						
MLD (Gy)	18.94 (7.14)	15.97 (6.93)	15.03 (6.96)	0.61	0.95	0.44
V_5_	57% (15%)	44% (18%)	40% (18%)	0.00	0.00	0.00
V_10_	49% (16%)	38% (16%)	36% (16%)	0.00	0.01	0.00
V_20_	38% (17%)	30% (13%)	29% (14%)	0.00	0.31	0.00
Contralateral lung						
MLD (Gy)	6.41 (3.51)	1.92 (2.62)	1.91 (2.48)	0.01	1.00	0.01
V_5_	45% (22%)	8% (9%)	7% (9%)	0.00	0.25	0.00
V_10_	22% (19%)	6% (8%)	6% (7%)	0.00	0.81	0.00
V_20_	6% (7%)	3% (5%)	4% (5%)	0.03	1.00	0.03

*p*_1,2_ is VMAT vs. IMPT; *p*_2,3_ is IMPT vs. IMCIT; *p*_1,3_ is VMAT vs. IMCIT

### Dose to the heart

**Fig. 2:**
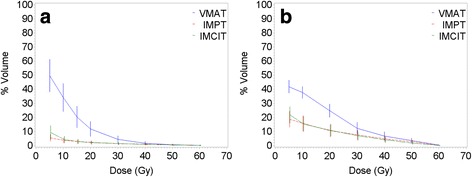
Dose distribution for the heart and the esophagus. **a** Dose distribution for the heart. **b** Dose distribution for the esophagus

When compared with VMAT, IMCIT resulted in significantly lower MHD, the D_1cc_ and the D_5cc_ (Table 4), and heart’s V_5_ – V_20_ (*p* < 0.05), (Fig. 2a, Additional file 2: Table S2). Similarly, IMPT led to significantly lower MHD and V_5_ – V_20_ than VMAT (*p* < 0.05). However, no significant difference in heart sparing was observed between IMCIT and IMPT.

**Table 4:** Selected dose parameters for other OARs

	VMAT_1_	IMPT_2_	IMCIT_3_		*p* value	
Parameters (Gy)		Mean (SD)		*p*_1,2_	*p*_2,3_	*p*_1,3_
*Heart*						
Mean dose	8.68 (7.13)	1.34 (1.99)	1.92 (2.96)	0.00	0.32	0.00
D_max_	45.53 (19.70)	41.71 (26.36)	40.31 (27.77)	0.38	0.49	0.28
D_0.01cc_	45.06 (19.74)	40.16 (26.95)	38.95 (28.28)	0.23	0.37	0.19
D_1cc_	38.89 (19.56)	32.97 (26.96)	31.79 (26.24)	0.19	0.13	0.05
D_5cc_	31.94 (16.96)	24.33 (22.75)	22.53 (21.57)	0.08	0.13	0.00
V_5_ (%)	50% (37%)	6% (8%)	9% (16%)	0.00	0.56	0.00
V_30_ (%)	4% (8%)	1% (2%)	1% (2%)	0.13	1.00	0.13
*Esophagus*						
Mean dose	11.37 (5.78)	5.37 (5.94)	5.73 (5.35)	0.00	0.23	0.00
D_max_	44.68 (15.88)	36.00 (22.10)	35.82 (22.93)	0.00	1.00	0.01
D_0.01cc_	44.18 (15.94)	36.32 (22.43)	34.42 (23.34)	0.03	0.70	0.00
D_1cc_	39.18 (16.33)	26.65 (25.26)	27.07 (23.59)	0.00	0.77	0.00
D_5cc_	31.20 (14.57)	17.91 (20.50)	17.79 (16.77)	0.00	1.00	0.00
*Spinal cord*						
Mean dose	8.32 (4.55)	3.18 (3.35)	3.25 (3.48)	0.00	1.00	0.00
D_max_	30.58 (8.87)	20.86 (11.38)	18.35 (12.39)	0.00	0.56	0.00
D_0.01cc_	29.74 (8.47)	19.05 (10.68)	16.86 (11.39)	0.00	0.38	0.00
D_1cc_	25.90 (8.22)	12.70 (9.76)	11.03 (9.93)	0.00	0.28	0.00
D_5cc_	21.57 (8.58)	8.68 (9.48)	8.54 (9.61)	0.00	0.92	0.00
*Major vessels*						
Mean dose	34.31 (6.81)	19.43 (9.84)	19.38 (9.63)	0.01	0.74	0.01
D_max_	63.27 (0.80)	62.37 (0.85)	62.14 (1.12)	0.01	0.48	0.02
D_0.01cc_	62.95 (0.78)	61.64 (0.97)	61.33 (1.12)	0.01	0.11	0.01
D_1cc_	60.64 (1.87)	58.23 (3.70)	57.30 (4.22)	0.001	0.01	0.01
D_5cc_	56.34 (5.75)	50.43 (12.22)	48.99 (12.83)	0.01	0.01	0.01
*Major airway*						
Mean dose	22.75 (16.90)	16.11 (17.00)	16.23 (16.70)	0.00	1.00	0.00
D_max_	43.21 (25.95)	43.53 (26.55)	42.64 (26.94)	0.77	0.19	0.56
D_0.01cc_	42.85 (25.90)	42.75 (26.20)	41.87 (26.80)	0.23	0.23	0.38
D_1cc_	38.96 (25.30)	36.37 (26.70)	36.18 (26.70)	0.02	0.43	0.03
D_5cc_	30.72 (21.57)	24.77 (24.98)	24.77 (24.19)	0.02	0.74	0.02

*p*_1,2_ is VMAT vs. IMPT; *p*_2,3_ is IMPT vs. IMCIT; *p*_1,3_ is VMAT vs. IMCIT

### Dose to the esophagus

Although no significant differences between IMPT and IMCIT was found, both led to significantly improved esophageal sparing, as compared with VMAT. The mean dose, D_max_, D_0.01cc_, D_1cc_, D_5cc_, and the V_5_ - V_30_ were significantly lower with PT (*p* < 0.05). These findings are shown in Table 4, Additional file 2: Table S2, and Fig. 2b.

### Dose to other thoracic organs

Dose reduction for the spinal cord was observed with PT for all dose parameters assessed (Table 4). However, no significant difference in spinal cord sparing was observed between IMPT and IMCIT. Similar findings were observed for the major blood vessels in the vicinity of the PTV. IMCIT further decreased the D_1cc_ and D_5cc_ to levels below those achieved with IMPT (*p* = 0.01). For major airway sparing, no significant difference between IMPT and IMCIT was observed, whereas PT, compared with VMAT, resulted in significantly lower mean doses, D_1cc_ and D_5cc_ for major airways (Table 4).

### Dose evaluation with recalculation on selected 4D phases for carbon ion and proton plans

Selected dose parameters for the PTV and the common OARs after dose recalculation with dose summation from 4D phases within the gating window, in comparison to those obtained from 3D planning, are shown in Additional file 2: Table 3. No statistically significant differences in the OAR dose parameters was observed. Among the 6 dose parameters assessed for the PTV (V_95_, D_max_, D_min_, CI, HI, nCI), dose recalculation decreased D_max_ and HI within the PTV (*p* < 0.05).

## Discussion

To the best of our knowledge, this study is the first study comparing photon VMAT with IMPT and IMCIT in the definitive treatment of loco-regionally confined lung tumors with hypo-fractionated radiotherapy. Although their clinical significance remains to be determined, our results indicated that fixed-beam IMCIT, compared with fixed-beam IMPT, may improve dose distribution for the tumor target, an effect possibly related to the sharper lateral dose gradient associated with carbon ions. The target volume dose coverage between VMAT and fixed-beam IMCIT was similar. Although the D_95_ was slightly improved with IMCIT compared with VMAT, the difference may be too small to have any clinical impact. However, fixed-beam IMCIT with 3 fields is greatly limited by the number and angles of beams that can be used to generate the most conformal treatment plan. In contrast, VMAT is one of the most mature and sophisticated forms of intensity-modulated photon therapy, and it can generate excellent dose conformity to the tumor target [11]. This difference indicates the great potential for IMCIT to further improve the dose distribution and the clinical efficacy of thoracic radiotherapy as it evolves in the future, given its known advantages over photons and protons (sharper lateral dose gradient, higher relative biological effectiveness, lower oxygen enhancement ratio, and more densely ionizing tracks that can lead to increased tumor DNA damage, less cell cycle dependence, and a stronger immunological response) [13, 14].

Suboptimal dose conformity due to the limitations of fixed-beam PT appears to be more prominent with IMPT, because protons, compared with carbon ions, are associated with greater lateral penumbra. Improving dose conformity for IMPT is important, because it may decrease the risk of radiation pneumonitis [31]. Although IMPT and CIRT, compared with 3D–CRT, have been shown to improve target volume coverage when multiple fields are used, IMRT has been shown to have better dose conformity and homogeneity than passively scattered PT [17–22]. Our study comparing IMPT and VMAT demonstrated similar findings. IMPT arcs may lead to better dose conformity than VMAT at the cost of increased dose heterogeneity, a finding that warrants further investigation [20].

PT has traditionally been shown to decrease the low dose volumes in the lungs [15–22]. IMPT, and especially IMPT arcs, compared with passively scattered PT, may further decrease the normal lung dose [16, 17, 19, 20]. Despite prominent low dose sparing, either passively scattered PT or IMPT may lead to higher volumes of the normal lung receiving doses >50% of the prescribed dose than photon therapy delivered with 3D–CRT or IMRT [17, 18]. However, this unique phenomenon of proton therapy has not been previously observed in comparisons of CIRT with 3D–CRT [21, 22]. Both IMPT and IMCIT significantly lowered the normal lung V_5_ - V_20_ in this study. This result was similar to those from previous studies comparing proton and photon therapies [15–20]. In contrast, VMAT, compared with either IMPT or IMCIT, appears to be associated with slightly lower but clinically non-significant volumes of normal lungs receiving high doses close to the prescribed dose, probably because of the suboptimal dose conformity associated with fixed-beam particle therapy (Fig. 1). In addition, IMCIT, compared with IMPT, may further decrease the ipsilateral lung volumes receiving low doses, thus leading to reduced total lung volumes receiving low doses. These findings may be associated with both the physical properties of particle beam therapy and the better dose conformity observed with VMAT and IMCIT compared with IMPT. However, improved normal lung sparing with IMCIT compared with VMAT was not found to be as dramatic as previously observed when passively scattered CIRT was compared with 3D–CRT, possibly because of the better control of normal lung dose associated with VMAT compared with 3D–CRT [21, 22]. Given PT’s physical properties, this sparing may be improved with arc-based methods of beam delivery [20].

Significant sparing of the heart, esophagus, and other thoracic OARs was observed with PT compared with VMAT (Table 4, Fig. 2). This finding was consistent with those of previous studies comparing PT and photon therapy [15–22]. Although no significant difference between IMCIT and IMPT was observed in the sparing of the heart, the esophagus, the spinal cord, or the major airway, the sparing of the heart and the major blood vessels in the high dose volumes may be best achieved with IMCIT. Overall, our findings suggested that although they are delivered with a limited number of beams and beams angles, fixed-beam IMPT and IMCIT may have an OAR sparing advantage over photon VMAT, which is more prominent with IMCIT than IMPT in the sparing of normal lungs and major blood vessels, owing to its improved dose conformity. Such an advantage may be further augmented as PT is more developed in the future.

Intensity modulation through active scanning may further improve dose conformity and OAR sparing in the thorax [16, 17, 19, 20]. This advantage is significantly limited by organ motion due to changes in radiological path length as a result of organ motion adjacent to the tumor, inter-field motion, and the interplay of interference between beam and tumor motion, which may result in tumor under-dose and OAR over-dose [32]. The interplay effect has been shown to significantly deteriorate target dose coverage and dose homogeneity when the motion amplitude is greater than 8 mm [33]. The correlation between motion amplitude and dose heterogeneity due to interplay, which leads to increased under-dosing within the target volume, has been further demonstrated in a 4D Monte Carlo simulation [34]. Such under-dosing has been modeled to significantly decrease the 2-year local control. However, this problem may be mitigated through fractionation, which leads to a motion-averaging effect on dose distribution, and the selection of larger spot size for the scanning beam. Together, these strategies can retain dose homogeneity with motion amplitudes of <20 mm. The mitigating effect of fractionation on interplay has also been shown in a series of 11 patients with stage III NSCLC for whom conventional fractionation with 35 fractions and hypo-fractionation with 10 fractions were modeled [35]. These observations further support our utilization of a hypo-fractionated schedule in the current study.

Respiratory motion is mainly managed through rescanning, gating, and beam tracking [32, 36, 37]. Rescanning mitigates the interplay effect by averaging the under-dosing and over-dosing patterns of the dose distribution. This process can be accomplished through volumetric or energy-slice by energy-slice rescanning while motion parameters are kept different between rescans. Rescanning mitigates the interplay effects without mitigating the target motion. Thus, adequate margins need to be maintained for adequate target dose coverage, possibly leading to suboptimal OAR sparing in conjunction with the dose blurring at the field borders. In addition, rescanning may be beneficial only when significant interplay exists under moderate to large respiratory motion [38]. Gating, which is already in clinical use for photon therapy to minimize respiratory motion-related dose degradation and unnecessary OAR irradiation, has been a major approach of respiratory motion management for particle therapy to minimize interplay. Through 4D dose calculation, adequate target volume dose coverage and dose homogeneity have been demonstrated with a gating window (GW) of ≤5 mm, and lung dose can be further decreased within the clinically acceptable range with shorter GWs up to 1 mm for spot scanning proton therapy [39]. This process supports our motion selection criteria of limiting the cranio-caudal motion to ≤5 mm through selecting the motion phases, including the maximal expiratory phase, by visual inspection of each patient’s 4D CT. Although 4D dose calculation to better account for interplay was not performed in this study, dose heterogeneities due to interplay for target motion of ≤5 mm were found to be within the clinically acceptable 5% for both carbon ions and protons at our institution (Additional file 1). How to best account for interplay in thoracic particle therapy will be more thoroughly assessed in future studies. A limitation for evaluating the effects of range uncertainties and interplay in this study is that errors may exist in the correlation between surrogate marker motion and internal tumor motion, and irregularities in the breathing pattern may also be present during real-time treatment. Such errors may be further decreased through periodical stereoscopic imaging intra-fractionally and phase-controlled rescanning (PCR) combined with gating for fast scanning particle beams [40]. Excellent dose conformity and enhanced OAR sparing have been demonstrated for PCR combined with gating [41]. However, rescanning during gating may not be necessary in all cases, especially when fractionated treatments are delivered, as suggested by the findings discussed above [34, 35, 38]. To account for irregular breathing patterns throughout the entire course of fractionated particle therapy, amplitude-based gating based on tumor location observed in 4D CT datasets has been adopted and routinely used clinically [42]. Although active tumor tracking may lead to the smallest high dose volume, this approach places a high demand on scanning speed, which must allow for rapid alteration of beam energy to adjust the Bragg peak in depth in relation to tumor motion [43]. This longitudinal compensation may be achieved through the use of wedges [44, 45]. However, tumor tracking remains an area of active research in thoracic particle therapy.

Owing to the scanning beam’s sensitivity to motion, 4D dose calculation for thoracic particle therapy has been advocated. The temporal density changes due to respiratory motion, which result in range uncertainties, can be incorporated into the treatment planning process. This process leads to decreased interplay and more robust treatment plans that, in contrast to 3D planning, can avoid unexpected under-dosing [46, 47]. Robust 4D planning is preferred for thoracic particle therapy, because dose errors in 3D planning are not always dependent on motion [47, 48]. However, this approach can be labor intensive and technically demanding. Thus, 3D planning with adequate motion management is still an acceptable approach in clinical practice [48]. No clinically significant differences in most of the commonly evaluated dose parameters were observed in our dose recalculation. This finding may be due to the limiting of respiratory motion in this selected group of patients, because the primary goal of the study was to compare the dose distribution among photon VMAT, IMCIT, and IMPT with minimal influence from respiratory motion. Therefore, 4D planning may have a more significant impact when significant respiratory motion is encountered. Our 4D dose evaluation is only a limited approximation of the actual dose, owing to a lack of full consideration of the interplay between respiratory motion and the dynamic beam scanning, which could be achieved only with more robust approaches for 4D dose calculation that are not commercially available currently [29, 30, 46, 47]. As a result, the limited dose variation observed after 4D dose evaluation, especially for the OAR dose parameters, may not fully capture the interplay effect and potentially be missing larger than actually observed dose uncertainties. This issue is also the major limitation of our study. Nevertheless, this study represents a step forward from 3D planning in accounting for range uncertainties that may lead to an overall decreased interplay effect [47]. The best approaches to 4D dose calculation and how to fully account for the interplay effect are beyond the scope of the current study. Robust 4D dose calculation for scanning beam particle therapy should be further assessed, and 4D planning should be considered whenever feasible, owing to the lack of direct correlation between motion amplitude and the interplay [29, 30, 47].

Another major limitation of this study is that fixed-angle particle beams are used. Dose distribution may be further improved with gantry-based systems through augmenting the known advantages of active scanning particle therapy with the increased number of angles for beam delivery. This question remains to be investigated in the future.

## Conclusion

In comparison with VMAT, fixed-beam IMCIT led to comparable dose conformity under limited respiratory motion. However, IMCIT had significantly better tumor target dose coverage and conformity than did IMPT. Although both IMPT and IMCIT led to significantly better thoracic OAR sparing than VMAT, IMCIT may further improve normal lung and major blood vessel sparing, as compared with IMPT.

## Additional files

Additional file 1: Measurement of dose heterogeneity for particle therapy. (DOC 27 kb)

Additional file 2: Table S1. Normal lung dose distribution. **Table S2.** Dose distribution for the heart and the esophagus. **Table S3.** Recalculated 4D (doses accumulated from 4D CT phases within the gating window) vs. 3D dose comparison for selected dose parameters for intensity modulated a) carbon ion therapy, and b) proton therapy with the Wilcoxon rank test. (DOC 114 kb)

## Abbreviations

3D–CRT: Three-dimensional conformal radiotherapy
AAA: Anisotropic analytical algorithm
CI: Conformity index
CIRT: Carbon ion radiotherapy
CTV: Clinical target volume
D_95_ & D_99_: Dose covering 95% and 99% of the volume, respectively
D_max_: Maximum dose
D_mean_: Mean dose
D_min_: Minimum dose
D_ncc_: Dose to n cc of the volume
D_Rx_: Prescribed dose
FWHM: Full-width half-maximum
GTV: Gross tumor volume
HI: Heterogeneity index
HMD: Heart’s mean dose
IG-IMRT: Image-guided and intensity-modulated radiotherapy
iGTV: Internal GTV
IMCIT: Intensity-modulated carbon ion therapy
IMPT: Intensity-modulated proton therapy
IMRT: Intensity-modulated radiotherapy
LEM: Local effect model
MFO: Multi-field optimization
MIP: Maximal intensity projection
MLD: Mean lung dose
nCI: New conformity index
NSCLC: Non-small cell lung cancer
OARs: Organs at risk
PCR: Phase controlled rescanning
PRO: Progressive resolution optimizer
PT: Particle therapy
PTV: Planning target volume
RA: Rapid arc
RBE: Radiobiological effectiveness
SBRT: Stereotactic body radiation therapy
V_95_ & V_99_: Volume receiving at least 95% and 99% of the prescribed dose, respectively
V_95%Rx_ and V_PTV_: The volume of tissue receiving ≥95% of the prescribed dose and the volume of the PTV, respectively
VMAT: Volumetric modulated arc therapy
V_n_: % of a volume receiving n Gy
