# Supplementary material for: Comparison of photon volumetric modulated arc therapy, intensity-modulated proton therapy, and intensity-modulated carbon ion therapy for delivery of hypo-fractionated thoracic radiotherapy
Source: Radiat Oncol. 2017 Aug 15;12:132. doi: 10.1186/s13014-017-0866-0 (PMC5558745; doi:10.1186/s13014-017-0866-0)
Supplement: Supplementary file 1 — Measurement of dose heterogeneity for particle therapy. (DOC 27 kb) [file 13014_2017_866_MOESM1_ESM.doc]

**Additional file 1**

For moving targets irradiated with active scanning beams, the threshold of tumor displacement was set to be within 5 mm in our motion-management protocol for lung-tumor treatments according to measured dose heterogeneity with measurements described here. Each measurement was performed with an individual EBT3 film that was sandwiched between two 1-cm thick PMMA slots with a cross section of 10 cm^2^ for each slot. The moving target, which consists of the two PMMA slots, was mounted on a moving phantom with its 2-cm thickness aligned with the beam’s central axis. The 10-cm^2^ surface of the irradiated film was oriented to be perpendicular to the beam’s central axis, and to be aligned with the direction of target movement (the cranio-caudal direction within a patient). A 5-cm uniform scanning field was used to irradiate the films at the center of the moving target. Although the dose response of an EBT3 film can vary up to 4-8% between batches that were made at different times, the variation in dose response of irradiated areas over a 10 cm x 10 cm square on the same film is usually within 2%. A flat-bed film scanner was also used to scan irradiated films to avoid any pitching effect caused by a film roller.

The Anzai system was used to gate the tumor movement by attaching an Anzai belt to the deformable phantom used for the current measurements. The deformable phantom allows changes in the external contour induced by an air bladder, which are synchronized with 1-dimensional (1D) translational target movement. Instead of moving the target within the deformable phantom, the 1D translation mechanism was used to move the target between two flat solid-water slots. The magnitude of sinusoidal target movement was set by a software controller. A gating window was set to deliver the beam at only half phase of each sinusoidal movement. Measurements with the applied gating window on motion magnitudes from 3.5 mm to 8.3 mm were performed with a physical dose of 2 Gy for both carbon-ion and proton beams. The frequency of target motion was also set to be 10 cycles per minute to simulate the frequency of normal breathing (10 to 15 respiratory cycles per minute). Measurement depth of the target was set at an 8-cm PMMA depth with in-air spot sizes of 4.1 - 5.5 mm and 11.1 - 13.8 mm full-width-half-maximum widths for carbon-ion and proton beams, respectively. For target motion amplitudes of 3.5 mm, 4.4 mm, 5.9 mm and 8.3 mm, measured dose heterogeneities were 4.5%, 3.8%, 5.6% and 6.25% for carbon ions; and 2.3%, 2.4%, 2.5%, and 2.3% for protons. The dose heterogeneity was only 3.5% for an additional measurement with a motion amplitude of 11.4 mm for protons. Although the dose heterogeneity due to larger than 5 mm target motion was found to be smaller than the clinically acceptable 5% for large in-air spot size protons, the threshold of target motion within a gating window according to the acquired 4D CT datasets was set to be 5.0 mm for clinical treatment of lung tumors at our institution for both types of particles.
