# Supplementary material for: Comparison of photon volumetric modulated arc therapy, intensity-modulated proton therapy, and intensity-modulated carbon ion therapy for delivery of hypo-fractionated thoracic radiotherapy
Source: Radiat Oncol. 2017 Aug 15;12:132. doi: 10.1186/s13014-017-0866-0 (PMC5558745; doi:10.1186/s13014-017-0866-0)
Supplement: Supplementary file 2 — Normal lung dose distribution. Table S2. Dose distribution for the heart and the esophagus. Table S3. Recalculated 4D (doses accumulated from 4D CT phases within the gating window) vs. 3D dose comparison for selected dose parameters for intensity modulated a) carbon ion therapy, and b) proton therapy with the Wilcoxon rank test. (DOC 114 kb) [file 13014_2017_866_MOESM2_ESM.doc]

**Table S1 Normal lung dose distribution**

| Parameters | VMAT_1_ | IMPT_2_ | IMCIT_3_ |  | *p* value |  |
| --- | --- | --- | --- | --- | --- | --- |
|  |  | Mean (SD) |  | *p*_1,2_ | *p*_2,3_ | *p*_1,3_ |
| Total lung - iGTV |  |  |  |  |  |  |
| V_5_ | 49% (18%) | 24% (11%) | 21% (12%) | 0.00 | 0.00 | 0.00 |
| V_10_ | 32% (16%) | 20% (10%) | 18% (10%) | 0.00 | 0.04 | 0.00 |
| V_20_ | 19% (10%) | 14% (7%) | 14% (8%) | 0.00 | 0.56 | 0.00 |
| V_30_ | 12% (7%) | 11% (5%) | 11% (6%) | 0.00 | 0.56 | 0.00 |
| V_40_ | 8% (4%) | 8% (4%) | 8% (4%) | 0.44 | 0.41 | 1.00 |
| V_50_ | 5% (3%) | 6% (3%) | 6% (3%) | 0.02 | 0.22 | 0.03 |
| V_60_ | 3% (1%) | 4% (2%) | 4% (2%) | 0.31 | N/A | 0.25 |
| Ipsilateral lung |  |  |  |  |  |  |
| V_5_ | 57% (15%) | 44% (18%) | 40% (18%) | 0.00 | 0.00 | 0.00 |
| V_10_ | 49% (16%) | 38% (16%) | 36% (16%) | 0.00 | 0.01 | 0.00 |
| V_20_ | 38% (17%) | 30% (13%) | 29% (14%) | 0.00 | 0.31 | 0.00 |
| V_30_ | 27% (14%) | 24% (11%) | 23% (11%) | 0.04 | 0.09 | 0.00 |
| V_40_ | 19% (10%) | 20% (10%) | 18% (10%) | 0.22 | 0.14 | 0.47 |
| V_50_ | 14% (8%) | 16% (9%) | 15% (9%) | 0.01 | 0.12 | 0.03 |
| V_60_ | 9% (6%) | 10% (7%) | 10% (7%) | 0.06 | 1.00 | 0.19 |
| Contralateral lung |  |  |  |  |  |  |
| V_5_ | 45% (22%) | 8% (9%) | 7% (9%) | 0.00 | 0.25 | 0.00 |
| V_10_ | 22% (19%) | 6% (8%) | 6% (7%) | 0.00 | 0.81 | 0.00 |
| V_20_ | 6% (7%) | 3% (5%) | 4% (5%) | 0.03 | 1.00 | 0.03 |
| V_30_ | 2% (3%) | 2% (4%) | 2% (4%) | 1.00 | 1.00 | 0.63 |
| V_40_ | 1% (2%) | 1% (2%) | 1% (2%) | 0.25 | 0.50 | 1.00 |
| V_50_ | 1% (1%) | 1% (2%) | 1% (2%) | 1.00 | 1.00 | 0.50 |
| V_60_ | 0% (1%) | 0% (1%) | 0% (1%) | 1.00 | N/A | N/A |

*p*_1,2_ is VMAT vs. IMPT; *p*_2,3_ is IMPT vs. IMCIT; *p*_1,3_ is VMAT vs. IMCIT.

**Table S2** **Dose volume distribution for the heart and the esophagus**

| Parameters | VMAT_1_ | IMPT_2_ | IMCIT_3_ |  | *p* value |  |
| --- | --- | --- | --- | --- | --- | --- |
|  |  | Mean (SD) |  | *p*_1,2_ | *p*_2,3_ | *p*_1,3_ |
| Heart |  |  |  |  |  |  |
| V_5_ | 50% (37%) | 6% (8%) | 9% (16%) | 0.00 | 0.56 | 0.00 |
| V_10_ | 34% (32%) | 4% (5%) | 4% (7%) | 0.00 | 0.63 | 0.00 |
| V_20_ | 12% (17%) | 2% (3%) | 2% (4%) | 0.03 | 1.00 | 0.03 |
| V_30_ | 4% (8%) | 1% (2%) | 1% (2%) | 0.13 | 1.00 | 0.13 |
| V_40_ | 2% (4%) | 1% (2%) | 1% (2%) | 0.25 | N/A | 0.50 |
| V_50_ | 1% (2%) | 0% (1%) | 0% (1%) | 1.00 | N/A | 0.50 |
| V_60_ | 0% (1%) | 0% (1%) | 0% (1%) | 1.00 | N/A | N/A |
| Esophagus |  |  |  |  |  |  |
| V_5_ | 42% (15%) | 19% (18%) | 22% (18%) | 0.00 | 0.13 | 0.00 |
| V_10_ | 38% (14%) | 26% (17%) | 15% (17%) | 0.00 | 0.83 | 0.00 |
| V_20_ | 25% (15%) | 11% (14%) | 11% (13%) | 0.00 | 1.00 | 0.00 |
| V_30_ | 12% (13%) | 8% (11%) | 7% (11%) | 0.02 | 0.13 | 0.02 |
| V_40_ | 7% (9%) | 5% (7%) | 4% (6%) | 0.13 | 0.50 | 0.13 |
| V_50_ | 4% (5%) | 3% (4%) | 2% (3%) | 0.38 | 0.25 | 0.25 |
| V_60_ | 0% (1%) | 0% (0%) | 0% (0%) | 0.50 | N/A | 0.25 |

*p*_1,2_ is VMAT vs. IMPT; *p*_2,3_ is IMPT vs. IMCIT; *p*_1,3_ is VMAT vs. IMCIT.

**Table S3** **Recalculated 4D (doses accumulated from 4D CT phases within the gating window) vs. 3D dose comparison for selected dose parameters for intensity modulated a) carbon ion therapy, and b) proton therapy with the Wilcoxon rank test.**

|  | **Carbon ion** | |  | **Proton** | |  |
| --- | --- | --- | --- | --- | --- | --- |
|  | **4D** | **3D** |  | **4D** | **3D** |  |
|  | Mean (SD) | Mean (SD) | *p* value | Mean (SD) | Mean (SD) | *p* value |
| **PTV** |  |  |  |  |  |  |
| V_95_ (%) | 99.48 (0.42) | 99.68 (0.41) | 0.19 | 99.55 (0.22) | 99.40 (0.98) | 0.32 |
| D_max_ (GyE) | 63.54 (0.93) | 65.25 (0.78) | **< 0.001** | 64.04 (0.73) | 65.98 (1.14) | **< 0.001** |
| D_min_ (GyE) | 51.39 (2.97) | 51.13 (2.80) | 0.90 | 49.07 (3.08) | 46.18 (3.77) | 0.09 |
| CI | 1.26 (0.08) | 1.30 (0.09) | 0.23 | 1.36 ( 0.09) | 1.37 (0.11) | 0.65 |
| HI | 1.06 (0.02) | 1.09 (0.01) | **0.002** | 1.07 (0.01) | 1.10 (0.02) | **0.001** |
| nCI | 1.46 (0.84) | 1.47 (0.77) | 0.47 | 1.48 (0.80) | 1.53 (0.78) | 0.25 |
| **Total lung** |  |  |  |  |  |  |
| MLD (GyE) | 7.89 (4.04) | 7.25 (3.67) | 0.63 | 8.45 (4.16) | 7.64 (3.53) | 0.53 |
| V_5_ (%) | 22.47 (11.73) | 21.29 (11.45) | 0.68 | 24.75 (12.01) | 23.44 (11.41) | 0.68 |
| V_20_ (%) | 14.93 (7.98) | 13.96 (7.63) | 0.68 | 15.72 (8.09) | 14.21 (6.87) | 0.63 |
| **Heart** |  |  |  |  |  |  |
| D_max_ (GyE) | 39.05 (27.31) | 40.31 (27.77) | 0.39 | 40.13 (26.86) | 41.71 (26.36) | 0.44 |
| MHD (GyE) | 2.06 (3.17) | 1.92 (2.96) | 0.73 | 1.44 (2.17) | 1.34 (1.99) | 0.71 |
| V_5_ (%) | 9.88 (16.44) | 8.45 (15.10) | 0.91 | 5.89 (8.49) | 5.52 (7.59) | 0.85 |
| **Esophagus** |  |  |  |  |  |  |
| D_max_ (GyE) | 34.56 (23.02) | 18.35 (12.39) | 0.55 | 35.55 (22.27) | 36.00 (22.10) | 0.85 |
| **Spinal cord** |  |  |  |  |  |  |
| D_max_ (GyE) | 20.22 (13.55) | 18.35 (12.39) | 0.65 | 20.45 (11.24) | 20.86 (11.38) | 1.00 |

MHD: mean heart dose.
